# Association between estimated glucose disposal rate and sarcopenia in U.S. adults: a cross-sectional study based on NHANES 2011–2018

**DOI:** 10.1186/s13098-026-02173-5

**Published:** 2026-05-11

**Authors:** Hongyan He, Xiaoqian Hu, Yuechi Luo, Yongxin Wu, Yuan Gao, Jing Yu, Kang Luo, Qian Xiao

**Affiliations:** Department of Geriatrics, Laboratory of Research and Translation for Geriatric Diseases, The First Affiliated Hospital of Chongqing Medical University, Chongqing, 400016 China

**Keywords:** Sarcopenia, Estimated glucose disposal rate (eGDR), Insulin resistance

## Abstract

**Background:**

The estimated glucose disposal rate (eGDR) is a novel indicator of insulin resistance that reflects the body’s ability to process glucose. The association between eGDR and sarcopenia in adults remains unclear. This study investigated the relationship between eGDR and sarcopenia to support the improved clinical identification of the condition.

**Methods:**

We analysed data from the 2011 to 2018 cycles of the National Health and Nutrition Examination Survey (NHANES). The final analytical sample comprised 7,147 participants. After applying survey weights, the population was 49.5% male and 50.5% female. We first calculated the weighted prevalence of sarcopenia among U.S. adults. Participants were then stratified into four groups based on eGDR quartiles. A weighted multivariate logistic regression model assessed the association between eGDR and sarcopenia risk, whereas a restricted cubic spline (RCS) examined their dose-response relationship. Subgroup analyses with interaction tests verified the stability of this association, and mediation analysis identified potential factors underlying the relationship.

**Results:**

A total of 7147 adult participants were included in this study. The weighted prevalence of sarcopenia was 6.7%, and the proportion of sarcopenic obesity among patients with sarcopenia was 73.1%. Weighted multivariate logistic regression revealed an obvious inverse association between eGDR and sarcopenia. In the fully adjusted model, compared with the lowest eGDR quartile group, the adjusted odds ratios (95% confidence intervals) for sarcopenia in quartiles 2nd to 4th quartile groups were 0.70 (95% CI: 0.52, 0.95; *p* < 0.001), 0.35 (95% CI: 0.25, 0.50; *p* < 0.001), and 0.12 (95% CI: 0.08, 0.18; *p* < 0.001), respectively. Subgroup analyses and interaction tests indicated that this relationship was not affected by factors such as age, sex, race, marital status, education, smoking, or drinking. Mediation analysis confirmed the mediating roles of inflammatory indices in the association between eGDR and sarcopenia (*p* < 0.001).

**Conclusion:**

A negative relationship was observed between eGDR and sarcopenia prevalence. The inflammatory response may mediate this relationship. eGDR may serve as a potential biomarker for the early identification and diagnosis of sarcopenia.

## Introduction

Sarcopenia is a progressive metabolic disorder characterized by an accelerated loss of muscle mass and function [1–3]. Sarcopenia is strongly associated with an increased risk of adverse outcomes, such as falls, disability, frailty, and mortality. The prevalence of sarcopenia in the general population ranges from 10% to 27%, while severe sarcopenia affects 2% to 9% of individuals, depending on the diagnostic criteria applied [4]. Screening and assessment typically target the elderly, yet muscle loss commences much earlier, often in middle age. Research has shown that muscle mass and strength in adults begin to decline around the age of 30, with strength deteriorating more rapidly and lower limb muscle mass exhibiting a particularly pronounced loss [5–7]. This early decline can substantially diminish residual muscle mass and strength in later life. Consequently, the effective prevention of sarcopenia requires extending the duration of peak muscle mass and strength while decelerating their rate of decline. In 2025, the Asian Working Group for Sarcopenia (AWGS) updated the consensus, positioning muscle health as a lifelong priority and establishing diagnostic criteria for the non-elderly population for the first time. This revision redefines sarcopenia from a condition exclusive to aging into a public health concern relevant to all adults. It underscores the need to advance clinical screening to include middle-aged and younger adult stages. Within this framework, identifying biomarkers for the early detection of muscle health risks is of considerable importance [8].

The pathophysiology of sarcopenia involves multiple factors, including reduced protein synthesis due to insulin resistance, chronic inflammation, mitochondrial dysfunction, hormonal changes, and fatty infiltration [9–11]. At present, the hyperinsulinemic-euglycemic clamp method is considered the gold standard for measuring insulin resistance in individuals with type 2 diabetes [12–14]. However, due to its time-consuming and costly operational requirements, it is not suitable for large-scale implementation. eGDR serves as an economical and easily calculable indicator for assessing insulin resistance. By integrating parameters such as fasting blood glucose, blood lipids, and blood pressure, eGDR can more comprehensively reflect the metabolic status in response to these factors [15, 16]. Furthermore, as a novel indicator of insulin resistance, studies have demonstrated its strong association with the gold standard clamp technique [17]. Recent findings indicate that eGDR is a significant predictor for several conditions, including diabetesm [18], cardiovascular diseases [19–22], metabolic syndrome [23], and stroke [24, 25]. Nevertheless, few studies have discussed the relation between eGDR and sarcopenia, and the mechanisms underlying their relationship are still unclear. Using data from the general population in the National Health and Nutrition Examination Survey (NHANES), this research examines the relationship between eGDR and sarcopenia and further evaluates whether chronic low-grade inflammation mediates this association, thereby offering a scientific foundation for clinical decision-making.

## Materials and methods

### Study design and population

**Fig. 1:**
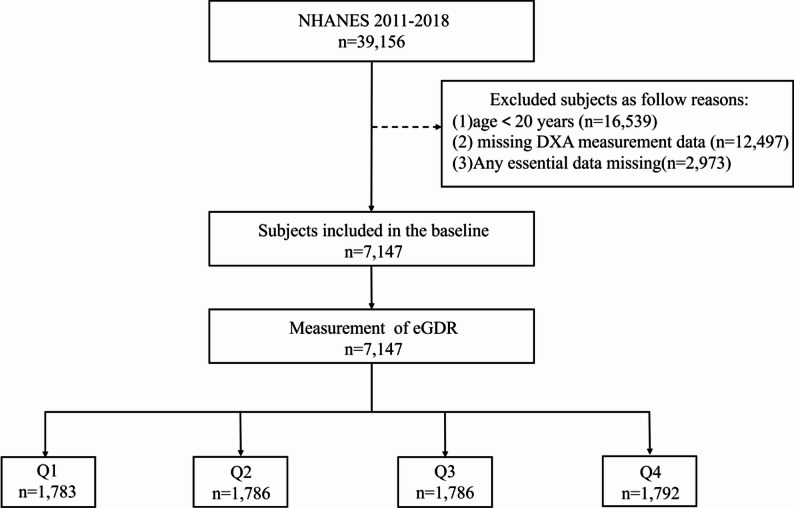
Flowchart of inclusion and exclusion of subjects. NHANES, the national health and nutrition examination survey; DXA, dual-energy X-ray absorptiometry

This study analyzed data from participants in NHANES between 2011 and 2018. Initiated in the early 1960s by the National Center for Health Statistics (NCHS), NHANES assesses the health and nutritional conditions of adults and children in the United States. A wide array of demographic, socioeconomic, dietary, and health information is collected by the survey. To ensure that the results were representative of the U.S. population, the survey data were weighted using the provided sampling weights. 32,009 individuals were excluded from the 39,156 surveyed participants between 2011 and 2018 according to the following criteria: (1) age < 20 years (*n* = 16,539); (2) missing DXA measurement data (*n* = 12,497); and (3) missing any essential data (*n* = 2,973). Therefore, 7,147 individuals were included in the final analysis, comprising 3,461 men and 3,686 women. Fig. 1 shows the detailed process for including and excluding participants. The weighted prevalence of sarcopenia was determined for U.S. adults aged 20 – 59 years. For the subsequent analysis, the estimated glucose disposal rate (eGDR) was categorised into quartiles. This study adhered to the Strengthening the Reporting of Observational Studies in Epidemiology (STROBE) guidelines.

### Data collection and definitions

Trained staff of the NHANES database conducted physical examinations and laboratory tests in accordance with established protocols. Participants were instructed to wear light clothing to measure body parameters, including height, weight, and waist circumference. Additionally, after resting quietly for five minutes, blood pressure was measured three to four times consecutively. Participants were required to fast for more than 9 h before blood sample collection. Serum samples were analyzed under uniform laboratory conditions to obtain data on complete blood count, liver and kidney function, electrolytes, lipid profile, and blood glucose.

The criteria for hypertension were as follows: the mean value of multiple blood pressure readings was calculated, and a systolic blood pressure ≥ 140 mmHg or a diastolic blood pressure ≥ 90 mmHg was considered indicative of hypertension. Individuals who either met these diagnostic criteria or reported a history of hypertension in the questionnaire were considered hypertensive. Diabetes mellitus was diagnosed via self-report in the questionnaire or a glycated hemoglobin (HbA1c) level higher than 6.5%.

### Sarcopenia and estimated glucose disposal rate (eGDR)

eGDR served as the primary exposure variable, and sarcopenia was the primary outcome variable in this study. Sarcopenia was diagnosed based on the criteria of the National Institutes of Health Foundation, using the ratio of appendicular skeletal muscle mass (ASM) to body mass index (BMI). The diagnostic thresholds were defined as ASM/BMI < 0.789 for men and < 0.512 for women. BMI was determined by dividing weight in kilograms by the square of height in meters. Muscle mass was determined using DXA, which helped determine the total appendicular skeletal muscle mass.

eGDR was determined using the following formula: eGDR (mg/kg/min) = 21.158 − (0.09 × WC) − (3.407 × hypertension) − (0.551 × HbA1c) [WC(cm), hypertension (yes = 1/no = 0), and HbA1c (%)].

### Other parameters and covariates

This study accounted for known or suspected confounders, including age, sex, race, education level, marital status, family income-to-poverty ratio (PIR), smoking, alcohol consumption, diabetes, sedentary time, albumin, creatinine, blood urea nitrogen, hemoglobin, white blood cell count, and metabolic syndrome-related indicators. We calculated the variance inflation factor (VIF) to evaluate the appropriateness of these covariates. The VIF values for all covariates ranged from 1.027 to 2.501, indicating no severe multicollinearity, as all values were below the conventional threshold of 5 (Table 1). Hypertension was excluded as a covariate from the correlation analysis because, as a binary variable, it is directly used in calculating the estimated glucose disposal rate (eGDR), creating significant collinearity. The basic demographic variables were defined as follows. Educational attainment was grouped into below high school, high school, and beyond high school. Marital status was classified as married (including those cohabiting or separated) and unmarried (including those who were widowed or divorced). The definition of smoking status is as follows: never smoking was named as “having smoked < 100 cigarettes in a lifetime”; former smoking was regarded as “having smoked > 100 cigarettes in a lifetime but having quit smoking”; and current smoking was regarded as “having smoked > 100 cigarettes in a lifetime and still smoking at the time of the interview.” The definition of alcohol consumption was as follows: never drinking was defined as “having consumed fewer than 12 alcoholic drinks in a lifetime”; former drinking was regarded as “having consumed ≥ 12 alcoholic drinks in one year but not in the past year, or having consumed > 12 alcoholic drinks in a lifetime but not in the previous year”; and current drinking was regarded as “consuming ≥ 12 alcoholic drinks per year and currently still drinking.”

**Table 1:** Variance inflation factor and tolerance

Term	VIF	VIF_CI_low	VIF_CI_high	SE_factor	Tolerance	Tolerance_CI_low	Tolerance_CI_high
Age	1.358689	1.321150	1.400614	1.165628	0.7360038	0.7139724	0.7569161
Gender	2.500859	2.411253	2.596155	1.581411	0.3998626	0.3851850	0.4147221
Race	1.545968	1.499743	1.596467	1.243369	0.6468441	0.6263829	0.6667808
Marry	1.092863	1.069153	1.124702	1.045401	0.9150280	0.8891245	0.9353200
Education	1.410061	1.370119	1.454313	1.187460	0.7091893	0.6876099	0.7298636
PIR	1.355339	1.317958	1.397114	1.164190	0.7378228	0.7157611	0.7587493
Smoking	1.356061	1.318646	1.397869	1.164500	0.7374300	0.7153748	0.7583534
Drink	1.187173	1.157986	1.221753	1.089575	0.8423369	0.8184961	0.8635682
Sedentary	1.027150	1.011076	1.066550	1.013484	0.9735678	0.9376029	0.9890451
Diabetes	1.814310	1.755827	1.877319	1.346963	0.5511737	0.5326745	0.5695323
Alb	1.366986	1.329058	1.409286	1.169182	0.7315362	0.7095792	0.7524124
BUN	1.259006	1.226225	1.296536	1.122054	0.7942776	0.7712860	0.8155107
Cr	2.002333	1.935307	2.074162	1.415038	0.4994174	0.4821223	0.5167139
UA	1.635977	1.585627	1.690656	1.279053	0.6112555	0.5914863	0.6306655
TG	1.379338	1.340831	1.422196	1.174452	0.7249854	0.7031382	0.7458061
HDL	1.465497	1.422982	1.512285	1.210577	0.6823624	0.6612510	0.7027495
Glu	1.808562	1.750340	1.871302	1.344828	0.5529254	0.5343873	0.5713175
Hb	1.925141	1.861619	1.993346	1.387494	0.5194425	0.5016691	0.5371668
WBC count	1.425119	1.384477	1.470058	1.193784	0.7016957	0.6802453	0.7222945
NLR	1.276263	1.242646	1.314538	1.129718	0.7835374	0.7607237	0.8047342
eGDR	1.670998	1.619046	1.727308	1.292671	0.5984449	0.5789354	0.6176475

### Statistical analysis

R 4.2.2 software was used to conduct the statistical analyses, with complex sampling weights applied to maintain the representativeness of the study population. First, eGDR values were stratified into quartiles, with the lowest quartile (Q1) as the reference group. The continuous variables in the baseline table are reported as means ± standard deviation (SD), and the categorical variables as counts (n) with weighted percentages (%). For complex survey samples, to provide a detailed characterization of the population, continuous variables were analyzed using t-tests and Wilcoxon rank-sum tests, whereas weighted percentages of categorical variables were evaluated using Rao–Scott chi-square tests. Initially, the relationship between eGDR and sarcopenia was determined through weighted multivariate logistic regression. To examine the dose–response relationship between eGDR and sarcopenia, a restricted cubic spline (RCS) analysis was performed. We evaluated the stability of the relationship through subgroup analyses and interaction tests. Finally, a mediation analysis was conducted to determine whether systemic inflammation, reflected by white blood cell and differential counts, mediated the association between eGDR and sarcopenia.

## Results

### Baseline characteristics

In this study, a total of 7,147 participants were included, which, after weighting, could represent 86,066,971 people in the U.S. population. Among them, the weighted proportion of men was 49.5%, and that of women was 50.5%. The weighted prevalence of sarcopenia was 6.7%, with a higher prevalence in men than in women (7.4% vs. 5.9%). The weighted prevalence of sarcopenia in young adults aged 20–39 years was 5.3%, and in middle-aged adults aged 40–59 years, it was 8.1%. Additionally, the weighted prevalence of sarcopenic obesity was approximately 4.9%, accounting for 73.1% of patients with sarcopenia. Participants were divided into four groups based on the quartiles of eGDR: Q1 < 6.57 mg/kg/min, 6.57 mg/kg/min ≤ Q2 < 9.10 mg/kg/min, 9.10 mg/kg/min ≤ Q3 < 10.40 mg/kg/min, and Q4 ≥ 10.40 mg/kg/min. Baseline characteristics indicated significant differences among the groups for most variables (*p* < 0.001) (Table 2). Age showed a decreasing trend with increasing eGDR. There was a significant difference in sex distribution, with the proportion of men decreasing from 58.2% to 37.3% and that of women increasing from 41.8% to 62.7% as eGDR gradually increased. The racial composition also showed significant differences, with non-Hispanic whites having a significantly higher proportion in the higher eGDR groups compared to other races. The prevalence of diabetes (20.5% to 0.3%), hypertension (87.3% to 0%), sarcopenia (13.5% to 1.0%), and sarcopenic obesity (11.5% to 0%) decreased significantly in the higher eGDR groups compared to other groups. Glucose, triglycerides, and uric acid showed a significant decreasing trend with increase of eGDR, and white blood cell count and NLR also showed a significant decrease with increasing eGDR.

**Table 2:** Baseline characteristics of the study population stratified by eGDR quartiles

Characteristic	Overall (*n* = 7,147)	eGDR				*p*-value
		Q1 (< 6.57) (*n* = 1,783)	Q2(6.57–9.10) (*n* = 1,786)	Q3(9.10–10.40) (*n* = 1,786)	Q4(≥ 10.40) (*n* = 1,792)	
Age, years, n(%)						< 0.001
< 40	3,618 (50.3)	508 (28.6)	847 (47.1)	967 (52.3)	1,296 (71.5)	
40–59	3,529 (49.7)	1,275 (71.4)	939 (52.9)	819 (47.7)	496 (28.5)	
Gender, n(%)						< 0.001
Male	3,461 (49.5)	938 (58.2)	892 (51.7)	916 (51.4)	715 (37.3)	
Female	3,686 (50.5)	845 (41.8)	894 (48.3)	870 (48.6)	1,077 (62.7)	
Race, n(%)						< 0.001
Non-Hispanic White	2,558 (61.8)	626 (60.6)	643 (60.6)	619 (61.8)	670 (64.1)	
Non-Hispanic Black	1,411 (10.7)	472 (14.6)	337 (10.2)	287 (8.6)	315 (9.9)	
Mexican American	1,103 (10.6)	276 (10.2)	347 (13.6)	323 (12.0)	157 (6.4)	
Other	2,075 (16.9)	409 (14.6)	459 (15.5)	557 (17.6)	650 (19.6)	
Education, n(%)						< 0.001
Below high school	1,220 (11.8)	336 (12.7)	326 (12.9)	333 (12.5)	225 (9.2)	
High school graduate/GED or equivalent	1,570 (21.6)	423 (24.2)	449 (25.5)	357 (19.1)	341 (17.8)	
High school or above	4,357 (66.6)	1,024 (63.1)	1,011 (61.6)	1,096 (68.4)	1,226 (73.0)	
Marry, n(%)						< 0.001
Married	4,595 (66.1)	1,204 (70.7)	1,196 (68.8)	1,219 (70.2)	976 (55.0)	
Unmarried	2,552 (33.9)	579 (29.3)	590 (31.2)	567 (29.8)	816 (45.0)	
PIR	2.97 ± 1.67	2.96 ± 1.65	2.84 ± 1.63	3.06 ± 1.69	3.03 ± 1.69	0.030
Sedentary (minutes)	416 ± 512	434 ± 487	431 ± 628	404 ± 462	398 ± 450	< 0.001
Smoking, n(%)						< 0.001
Never	4,328 (58.6)	969 (51.8)	1,030 (56.1)	1,133 (61.5)	1,196 (64.6)	
Previous	1,208 (19.7)	389 (25.3)	310 (19.7)	296 (19.3)	213 (15.0)	
Now	1,611 (21.6)	425 (22.9)	446 (24.2)	357 (19.3)	383 (20.4)	
Drink, n(%)						0.126
Never	735 (7.7)	190 (7.9)	208 (9.2)	170 (6.6)	167 (7.2)	
Previous	1,241 (13.7)	313 (14.9)	301 (12.6)	313 (14.5)	314 (12.8)	
Now	5,171 (78.6)	1,280 (77.2)	1,277 (78.2)	1,303 (79.0)	1,311 (80.0)	
Sarcopenia, n(%)						< 0.001
No	6,547 (93.3)	1,517 (86.5)	1,592 (91.0)	1,676 (96.2)	1,762 (99.0)	
Yes	600 (6.7)	266 (13.5)	194 (9.0)	110 (3.8)	30 (1.0)	
Sarcopenic Obesity, n(%)						< 0.001
No	6,742 (95.1)	1,562 (88.5)	1,628 (92.4)	1,760 (99.0)	1,792 (100.0)	
Yes	405 (4.9)	221 (11.5)	158 (7.6)	26 (1.0)	0 (0.0)	
Diabetes, n(%)						< 0.001
No	6,491 (93.2)	1,335 (79.5)	1,627 (94.3)	1,744 (98.1)	1,785 (99.7)	
Yes	656 (6.8)	448 (20.5)	159 (5.7)	42 (1.9)	7 (0.3)	
Hypertension, n(%)						< 0.001
No	5,242 (74.8)	224 (12.7)	1,440 (82.1)	1,786 (100.0)	1,792 (100.0)	
Yes	1,905 (25.2)	1,559 (87.3)	346 (17.9)	0 (0.0)	0 (0.0)	
Alb, g/L	43.2 ± 3.4	42.3 ± 3.5	42.7 ± 3.2	43.6 ± 3.2	44.0 ± 3.3	< 0.001
BUN, mmol/L	4.62 ± 1.53	4.91 ± 1.84	4.59 ± 1.44	4.61 ± 1.42	4.41 ± 1.36	< 0.001
Cr, umol/L	75 ± 27	79 ± 43	75 ± 23	76 ± 17	72 ± 15	< 0.001
UA, umol/L	314 ± 80	345 ± 83	327 ± 77	311 ± 75	275 ± 66	< 0.001
TG, mmol/L	1.70 ± 1.51	2.17 ± 1.53	1.92 ± 1.50	1.66 ± 1.84	1.07 ± 0.66	< 0.001
HDL, mmol/L	1.38 ± 0.42	1.22 ± 0.34	1.28 ± 0.38	1.39 ± 0.40	1.60 ± 0.43	< 0.001
Glu, mmol/L	5.36 ± 1.76	6.27 ± 3.00	5.32 ± 1.32	5.08 ± 0.75	4.83 ± 0.61	< 0.001
Hemoglobin, g/dL	14.25 ± 1.45	14.43 ± 1.54	14.33 ± 1.40	14.29 ± 1.44	13.97 ± 1.37	< 0.001
WBC count, 10^9^/L	7.40 ± 2.17	7.94 ± 2.29	7.67 ± 2.26	7.19 ± 1.99	6.83 ± 1.98	< 0.001
NLR	2.08 ± 1.00	2.17 ± 1.03	2.15 ± 1.08	2.01 ± 0.88	2.01 ± 0.98	< 0.001

eGDR, the estimated glucose disposal rate; PIR, ratio of family income to poverty; Alb, albumin; BUN, blood urea nitrogen; Cr, creatinine; UA, uric acid; TG, triglycerides; HDL, high-density lipoprotein; Glu, glucose; WBC, white blood cell; NLR, the neutrophil-to-lymphocyte ratio

### Relationship between sarcopenia and eGDR

#### Multivariate regression analysis

**Fig. 2:**
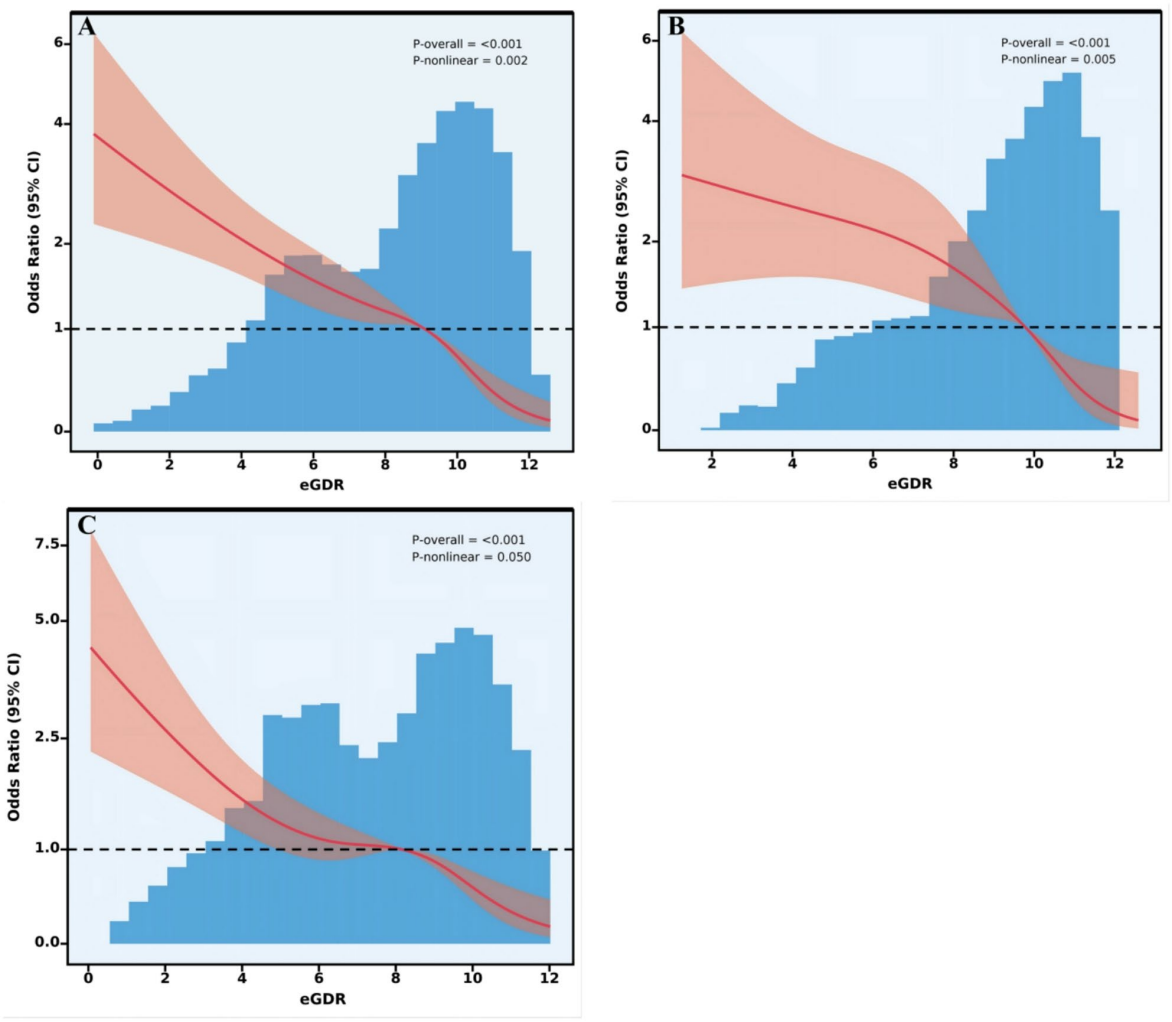
**(A)** RCS curves of eGDR values for each research subject related to sarcopenia. **(B)** RCS curves of eGDR values in the 20–39 age group related to sarcopenia. **(C)** RCS curves of eGDR values in the 40–59 age group related to sarcopenia

In this study, a weighted multiple logistic regression analysis was conducted to examine the association between eGDR and sarcopenia. Building on previous research, various covariates were included, and multiple models were developed incrementally. The results from the weighted multivariate logistic regression revealed that higher eGDR levels were significantly associated with a reduced risk of sarcopenia. As outlined in Table 3 , Model 1 is the unadjusted crude model, Model 2 includes demographic factors such as age, sex, race, marital status, education, and PIR, among others, while Model 3 further adjusts for age, sex, race, marital status, education, PIR, smoking, alcohol consumption, sedentary behaviour, diabetes, albumin, creatinine, urea nitrogen, hemoglobin, white blood cell count, and metabolic syndrome parameters. In the fully adjusted model (Model 3), individuals in the highest eGDR group (10.4–13.2 mg/kg/min) exhibited a sarcopenia risk that was merely 12% (OR = 0.12, 95% CI: 0.08–0.18) of that in the lowest group (-2.72–6.57 mg/kg/min). To investigate the potential dose–response relationship and ascertain whether the association is nonlinear, we utilized a restricted cubic spline (RCS) model with four knots located at the 5th, 35th, 65th, and 95th percentiles. The restricted cubic spline curves (RCS curves) presented in Figs. 2 A–C illustrate the dose–response relationship between these two variables. In the U.S. population aged 20–59 years, the association between eGDR and sarcopenia demonstrated a nonlinear negative correlation, with nonlinear p-values for each age group being ≤ 0.05 (Table 3).

**Table 3:** Weighted logistic regression model between eGDR and the risk of sarcopenia

Characteristic	Model 1			Model 2			Model 3		
	OR	95% CI	*p*-value	OR	95% CI	*p*-value	OR	95% CI	*p*-value
eGDR									
[-2.72,6.57)	—	—		—	—		—	—	
[6.57,9.1)	0.64	0.49, 0.82	< 0.001	0.60	0.46, 0.78	< 0.001	0.70	0.52, 0.95	< 0.001
[9.1,10.4)	0.25	0.18, 0.36	< 0.001	0.25	0.18, 0.35	< 0.001	0.35	0.25, 0.50	< 0.001
[10.4,13.2]	0.06	0.04, 0.09	< 0.001	0.07	0.05, 0.11	< 0.001	0.12	0.08, 0.18	< 0.001

Model 1: no covariates were adjusted

Model 2: adjusted for age, gender, race, education, marry, PIR

Mode 3: adjusted for age, gender, race, education, marry, PIR, diabetes, smoking, drink, sedentary, Alb, BUN, Cr, UA, TG, HDL, Glu, WBC, hemoglobin, NLR

#### Subgroup analysis and interaction tests

**Fig. 3:**
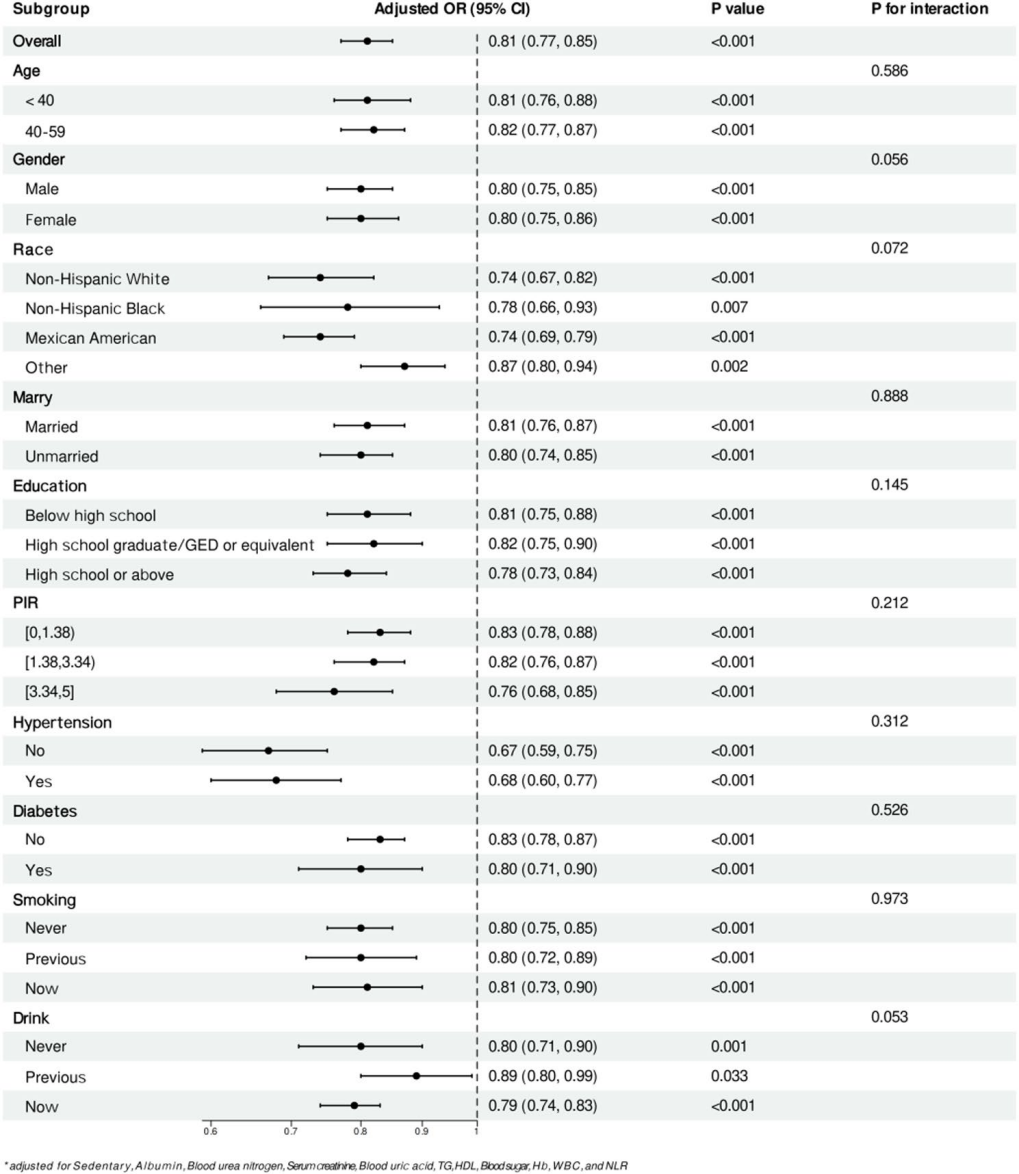
Subgroup analysis and interaction test results of eGDR and sarcopenia

To ensure the reliability of our findings, we performed subgroup analyses and interaction tests on the research data. After adjusting for potential confounding variables, it was determined that the association between eGDR and sarcopenia remained unaffected by variables, such as age, sex, race, marital status, educational level, economic status, hypertension, diabetes, smoking, and alcohol consumption. The interaction tests produced p-values exceeding 0.05, supporting the consistency of the results. The detailed findings are illustrated in Fig. 3.

#### Mediation analysis

To elucidate the mediating factors between eGDR and sarcopenia, we performed a mediation analysis, as presented in Table 4. After comprehensive adjustment for covariates, including age, sex, race, education, marital status, economic status, diabetes, sedentary time, smoking, drinking, albumin, serum creatinine, serum urea nitrogen, serum uric acid, triglycerides, high-density lipoprotein, and blood glucose, the findings revealed that white blood cell count, neutrophil count, lymphocyte count, and monocyte count exhibited significant indirect effects (*p* < 0.001). Notably, the mediation ratio of white blood cell count was the highest (7.9%, 95% CI: 4.2–11.0), whereas NLR did not demonstrate a significant mediating effect (*p* > 0.05). This indicates that systemic inflammatory levels, as reflected by white blood cells and their classification counts, mediate the relationship between eGDR and sarcopenia.

**Table 4:** The mediating role of white blood cells and their classification counts between eGDR

Mediator	Total effect		Indirect effect		Direct effect		Proportion mediated, % (95% CI)
	Coefficient (95% CI)	*P* value	Coefficient (95% CI)	*P* value	Coefficient (95% CI)	*P* value	
WBC count	-0.02998 (-0.03804, -0.02195)	< 0.001	-0.00236 (-0.00334, -0.00140)	< 0.001	-0.02762 (-0.03538, -0.01959)	< 0.001	7.9 (4.2, 11.0)
Neutrophils count	-0.02966 (-0.03756, -0.02149)	< 0.001	-0.00174 (-0.00255, -0.00094)	< 0.001	-0.02793 (-0.03584, -0.01977)	< 0.001	5.9 (2.8, 8.5)
Lymphocyte count	-0.03000 (-0.03786, -0.02182)	< 0.001	-0.00140 (-0.00219, -0.00070)	< 0.001	-0.02860 (-0.03654, -0.02044)	< 0.001	4.7 (2.3, 7.0)
Monocyte count	-0.03059 (-0.03867, -0.02222)	< 0.001	-0.00079 (-0.00150, -0.00010)	< 0.001	-0.02981 (-0.03794, -0.02145)	< 0.001	2.5 (0.3, 4.8)
NLR	-0.02961 (-0.03760, -0.02143)	< 0.001	-0.00013 (-0.00040, 0.00008)	0.240	-0.02947 (-0.03751, -0.02127)	< 0.001	0.4 (-0.3, 1.4)

WBC, white blood cell; NLR, the neutrophil-to-lymphocyte ratio

## Discussion

This study utilized nationally representative data from the National Health and Nutrition Examination Survey (NHANES) spanning 2011 to 2018 in the United States. Employing a weighted analysis method, the findings were extrapolated to represent a population of 86,066,971 individuals in the United States. The statistical outcomes revealed that the weighted prevalence of sarcopenia among individuals aged 20 to 59 years was approximately 6.7%, while the weighted prevalence of sarcopenic obesity was approximately 4.9%, constituting 73.1% of sarcopenia cases. Additionally, this study is the first to investigate the association between eGDR and sarcopenia in adult Americans. The results demonstrated that a reduction in eGDR levels was significantly correlated with an elevated risk of sarcopenia, independent of confounding variables, such as age, sex, race, educational attainment, marital status, and economic status. Further mediation analysis indicated that systemic inflammatory levels, as reflected by white blood cell counts and their classifications, partially mediated this relationship. This study provides a novel foundation for the clinical application of the simple indicator eGDR in the early identification of sarcopenia.

Sarcopenia is a condition marked by the gradual and extensive reduction in skeletal muscle mass and strength, which is closely associated with increased weakness, functional impairments, higher hospitalisation costs, and a greater risk of early mortality [26–28]. Historically, sarcopenia has primarily been associated with aging [29], with research largely focusing on the elderly. However, lifestyle changes have led to a growing incidence of sarcopenia among younger and middle-aged individuals. Studies utilizing various assessment methods across different ethnic groups have found that approximately 10% of young adults aged 20 to 30 years may be diagnosed with sarcopenia [30]. Epidemiological studies employing different sarcopenia evaluation techniques in various regions have highlighted the significant threat that sarcopenia poses to younger and middle-aged populations. In South Korea, a study of over 300,000 people found that approximately 15.5% of those aged 20 to 30 years had sarcopenia [31]. In Thailand, a study of 832 people showed that more than a quarter of men and women aged 30 to 40 years had sarcopenia, with the highest rates in the 50–60 age group [32]. In the U.S., data from 7,147 participants, representing 86,066,971 people, showed that approximately 6.7% of people aged 20 to 59 years had sarcopenia. In 2025, AWGS released an updated consensus, emphasizing the importance of continuous monitoring of skeletal muscle, a crucial organ for healthy longevity, from middle age to old age, with risk identification and management tailored to different life stages [8].

Elderly individuals often experience multiple chronic health conditions, whereas younger and middle-aged populations generally exhibit better overall health. This distinction suggests that the characteristics of sarcopenia differ between adults and the elderly. This study employed the World Health Organization’s criteria for obesity (body mass index [BMI] ≥ 30.0 kg/m²) in conjunction with the Foundation for the National Institutes of Health’s criteria for sarcopenia to conduct a statistical analysis of sarcopenic obesity prevalence within the study cohort. The analysis revealed a weighted prevalence of sarcopenic obesity at approximately 4.9%, accounting for 73.1% of individuals with sarcopenia. These findings suggest that obesity and related metabolic disorders may have a more pronounced impact on sarcopenia in younger and middle-aged populations. The global rise in obesity and overweight prevalence provides a broader context for this issue. Since 1980, the prevalence of overweight and obesity has increased significantly worldwide, with nearly one-third of the global population now classified as overweight or obese [33]. Numerous epidemiological studies have established a strong association between high body mass index (BMI) and chronic diseases, including cardiovascular diseases, diabetes, chronic kidney disease, various cancers, and musculoskeletal disorders. Being overweight has emerged as a significant risk factor for disease onset and mortality [34]. Given the growing burden of obesity, weight management has become a crucial aspect of health promotion. However, interventions aimed at weight control may inadvertently contribute to muscle loss. Methods such as dieting, pharmacotherapy, and surgical weight loss can lead to reductions in skeletal muscle mass or strength [35–37]. Urban environments, characterised by sedentary and unhealthy lifestyles, further exacerbate this issue. A study conducted in Thailand suggested that the impact of urban environments on sarcopenia may surpass that of BMI or age [32].

Sarcopenic obesity (SO) represents a distinct subtype of sarcopenia, primarily characterized by the concurrent presence of obesity and sarcopenia [38]. As a significant phenotype associated with metabolic dysfunction, inflammation, and functional decline, the pathogenesis of SO is notably complex, involving interactions between adipose tissue and skeletal muscle. In the context of obesity, adipocytes undergo hypertrophy, hyperplasia, and activation; pro-inflammatory macrophages and other immune cells accumulate; various adipokines are secreted aberrantly; and lipids are ectopically deposited within skeletal muscle. These intramuscular lipids and their derivatives induce mitochondrial dysfunction, promote lipotoxicity and insulin resistance, and further enhance the secretion of specific pro-inflammatory myokines, thereby precipitating muscle dysfunction. Conversely, these myokines exacerbate adipose tissue inflammation and promote systemic chronic low-grade inflammation, thus establishing a vicious cycle of adipose tissue inflammation and skeletal muscle damage [39]. Compared to simple obesity and sarcopenia, SO is more frequently associated with cardiovascular diseases, cancer, diabetes, fractures, and frailty, and is often linked to higher rates of hospitalization, disability, and mortality [40].

Sarcopenia is associated with compromised muscle repair and regeneration, primarily due to a reduction in muscle satellite cells, increased infiltration of inflammatory cells, oxidative stress, mitochondrial dysfunction, and insulin resistance, among other factors [41, 42]. The development of effective biomarkers could provide a straightforward, cost-effective, and efficient means of identifying individuals with sarcopenia [43]. While elderly individuals with sarcopenia often experience a decline in multiple system functions, younger and middle-aged individuals tend to exhibit more pronounced and isolated metabolic abnormalities. This presents a critical opportunity for early detection and intervention. In this context, the estimated glucose disposal rate (eGDR) serves as a comprehensive indicator of the body’s metabolic state, offering an alternative measure of insulin resistance with significant potential for the early identification of adult sarcopenia. Since its inception, eGDR has been utilized to evaluate insulin resistance in patients undergoing long-term insulin therapy for type 1 diabetes and has increasingly been applied to assess insulin resistance in patients with type 2 diabetes. Its reliability has been confirmed through comparisons with the gold standard clamp test in patients with type 2 diabetes [44]. Research indicates that insulin resistance is implicated in the pathogenesis of various chronic diseases, including cardiovascular diseases [19, 45, 46], non-alcoholic fatty liver diseas [46], and metabolic syndrome [47]. As a straightforward indicator of insulin resistance, eGDR demonstrates predictive efficacy for related diseases comparable to the HOMA index. A recent prospective cohort study by Zheng et al. demonstrated that eGDR is independently associated with the risk of cardiovascular disease (CVD). Individuals with persistently low or increasing eGDR levels within a low range are at a higher risk of CVD, underscoring the importance of dynamic eGDR monitoring for CVD prevention and management [48]. In a study examining the relationship between eGDR and metabolic syndrome (MetS), eGDR exhibited a significantly higher predictive ability for all-cause mortality in MetS patients compared to HOMA-IR and the TyG index [47]. Another study highlighted that eGDR has a stronger predictive efficacy for CVD events in patients with diabetes and prediabetes than other insulin resistance indicators, such as HOMA-IR, TyG, QUICKI, and METS-IR [49]. While there is a limited body of research on the association between eGDR and sarcopenia, this study investigated the relationship between this novel index of insulin resistance and sarcopenia. Utilizing a weighted multiple logistic regression model, we identified a significant inverse correlation between eGDR and sarcopenia. In the fully adjusted model, the adjusted risk ratios for sarcopenia in the second, third, and fourth quartiles of eGDR, compared to the lowest quartile, were 0.70 (95% CI: 0.52, 0.95; *p* < 0.001), 0.35 (95% CI: 0.25, 0.50; *p* < 0.001), and 0.12 (95% CI: 0.08, 0.18; *p* < 0.001), respectively. Importantly, subgroup analyses demonstrated that this association was consistent across different demographics, including age, sex, race, education, marital status, and economic status, underscoring the robustness of the findings. These results suggest that eGDR holds significant promise as a tool for the early identification of sarcopenia.

To further investigate potential indirect mediators of the relationship between eGDR and sarcopenia, we conducted a mediation analysis. Insulin resistance is strongly associated with a reduction in muscle mass [50, 51]. Our study suggests that the previously observed relationship between eGDR and sarcopenia may be mediated by the chronic inflammatory state of the body. In the context of aging or obesity, M1-type macrophages secrete pro-inflammatory factors, such as tumour necrosis factor α (TNF-α), which can directly activate Jun N-terminal kinase (JNK) and κB kinase (IKK) β inhibitors in target cells, such as skeletal muscle. This activation promotes the phosphorylation of serine sites on IRS-1, interfering with the normal tyrosine phosphorylation process of IRS-1, thereby blocking downstream PI3K/Akt signal transduction and ultimately reducing insulin-stimulated glucose uptake capacity [52, 53]. Furthermore, insulin resistance can exacerbate inflammation through multiple pathways. Inflammation and insulin resistance synergistically contribute to the development of sarcopenia. Total white blood cell count and differential white blood cell counts are considered among the most common systemic inflammatory biomarkers for the clinical assessment of inflammation and metabolic disorders. Increasing evidence supports the role of subclinical low-level inflammation in the pathogenesis of type 2 diabetes, with white blood cell count serving as an indicator of such inflammation [54], potentially elucidating the relationship between white blood cell count and insulin resistance. Prospective studies by Vozarova et al. and Zang et al. have demonstrated that an increase in total white blood cell count can predict the risk of impaired fasting glucose, reduced insulin sensitivity, and an elevated risk of type 2 diabetes [55, 56]. Although the precise pathophysiological mechanisms and etiology of low muscle mass remain unclear [57], research indicates that the inflammatory response may play a significant role in the development of sarcopenia. The decline in muscle mass and strength under various inflammatory conditions is linked to increased levels of pro-inflammatory cytokines, such as TNF-α, IL-1β, IL-6, and IFN-γ [58], which primarily induce muscle atrophy by enhancing protein degradation and inhibiting protein synthesis. A meta-analysis and a cross-sectional study have shown that elevated inflammatory biomarkers are associated with an increased risk of sarcopenia [59]. Additionally, studies utilizing the large sample database of the Korean National Health and Nutrition Survey have identified a correlation between white blood cell count and the risk of sarcopenia [60, 61]. Our study also indicates that the inflammatory response level mediates the relationship between insulin resistance and sarcopenia. Specifically, the relationship between eGDR, an indicator of insulin resistance, and sarcopenia is partially mediated by the systemic inflammatory level, as reflected by white blood cell count, neutrophil count, lymphocyte count, and monocyte count. This suggests that anti-inflammatory interventions may represent a potential strategy for ameliorating insulin resistance-related sarcopenia.

## Strengths and limitations

This study draws on data from the comprehensive NHANES database, which targets the general population. By applying weighted analysis to the extracted data, the conclusions are rendered more relevant. Initially, we conducted a statistical evaluation of the weighted prevalence of sarcopenia among American adults, identifying a prevalence rate of approximately 6.7%. Importantly, among individuals diagnosed with sarcopenia, nearly 73.1% also presented with sarcopenic obesity. This observation highlights that sarcopenia is not limited to older adults; rather, it indicates that in middle-aged and younger adults, sarcopenia may be heavily influenced by obesity and related metabolic disorders. With shifts in lifestyle trends, there is a growing risk of sarcopenia among younger populations. Therefore, we stress the urgent need for sarcopenia screening. Our study further reveals that in American adults, a reduction in eGDR is significantly linked to an increased risk of sarcopenia. The validity of these findings is reinforced by subgroup analyses and interaction tests. Moreover, we have, for the first time, confirmed the mediating role of systemic inflammatory response levels between eGDR and sarcopenia, providing a clinical perspective on the impact of inflammatory responses on insulin resistance and sarcopenia.

Nonetheless, this study has a few limitations. Primarily, as a cross-sectional study, it could not determine a causal link between eGDR and sarcopenia; further verification is needed through prospective cohort studies in the future. Considering that muscle strength is generally regarded as more clinically significant than muscle mass, it is imperative to elucidate the relationship between eGDR and muscle strength. However, owing to the absence of systematic grip strength data within the study population, this investigation was unable to conduct a comprehensive analysis of the relationship between eGDR and muscle strength as assessed by grip strength. In future research, we intend to further investigate the intrinsic association between metabolic indicators and muscle strength to enhance our understanding of adult sarcopenia. Moreover, although relevant covariates were included in the analysis based on previous studies, certain lipoprotein types, such as LDL, had excessive missing values in the NHANES database. Furthermore, medications, dietary habits, and lifestyle factors affecting the musculoskeletal system were not incorporated into the analysis. Thus, potential confounding factors could not be ruled out. In our additional mediation analysis, we identified the potential mediating influence of the inflammatory response level, as evidenced by white blood cell counts and their classifications, in the relationship between eGDR and sarcopenia. However, the lack of other inflammatory response factors in the NHANES database limits our ability to fully evaluate the significance of the inflammatory response level in the association between IR and sarcopenia. Moreover, the roles of oxidative stress, hormonal factors, and other variables remain unexplored. Therefore, further research is necessary to investigate the mediating effects of other inflammatory factors and additional variables in this context.

## Conclusion

This investigation, drawing on data from the NHANES database in the United States, marks the first exploration of the link between eGDR and sarcopenia in American adults. The study reveals that the weighted prevalence of sarcopenia among individuals aged 20–59 is approximately 6.7%, with sarcopenic obesity accounting for about 4.9%, which represents 73.1% of those with sarcopenia. The findings imply that obesity and associated metabolic disorders may play a significant role in the development of sarcopenia in middle-aged and younger adults, highlighting the necessity for early detection in this group. Moreover, our analysis demonstrates a significant inverse relationship between eGDR and the risk of sarcopenia, suggesting that lower eGDR values correlate with an increased risk of sarcopenia. The robustness of these findings is supported by subgroup analyses and interaction tests, which affirm an independent association between eGDR and sarcopenia. However, further prospective studies are required to confirm this relationship. Additionally, mediation analysis suggests that systemic inflammatory response levels, indicated by white blood cell counts and their classifications, may mediate the association between eGDR and sarcopenia. This insight provides a critical foundation for the clinical screening of sarcopenia, with eGDR potentially serving as a straightforward tool for identifying high-risk individuals. Continued research is essential to thoroughly investigate the causal pathways and underlying mechanisms linking insulin resistance and sarcopenia.

## Data Availability

Data are available from the NHANES (NHANES—National Health and Nutrition Examination Survey Homepage (cdc.gov)) in 2011–2018.
